# Supplementary material for: lncRNA-screen: an interactive platform for computationally screening long non-coding RNAs in large genomics datasets
Source: BMC Genomics. 2017 Jun 5;18:434. doi: 10.1186/s12864-017-3817-0 (PMC5458484; doi:10.1186/s12864-017-3817-0)
Supplement: Supplementary file 1 — Supplementary material 2. (DOCX 128 kb) [file 12864_2017_3817_MOESM1_ESM.docx]

**SUPPLEMENTARY MATERIAL 2**

**RNA-Seq Standard**

RNA-Seq Standard Pipeline is designed to perform standard RNA-Seq analysis for Illumina TruSeq sequencing data. It generates a standard RNA-Seq HTML report which includes alignment quality assessment, sample distance evaluation, PCA plots, dispersion plots, MA plots, raw count/normalized count/fpkm table, pairwise differential expression analysis, top differential expressed genes heatmap, user-defined targets differential expression analysis/heatmap.

**Setting up:**

1. You first need to have a [referenceFiles](https://github.com/NYU-BFX/RNA-Seq_Standard/blob/master/referenceFiles) folder containing pre-built [STAR reference](https://github.com/NYU-BFX/RNA-Seq_Standard/blob/master/referenceFiles/STAR_Reference), [chromInfo.txt](https://github.com/NYU-BFX/RNA-Seq_Standard/blob/master/referenceFiles/chromInfo.txt), etc. If you do not have it, you can run the [files_needed.bash](https://github.com/NYU-BFX/RNA-Seq_Standard/blob/master/referenceFiles/files_needed.bash) to download the necessary files and generate the STAR index. But you need to change "hg19" and "v19" in the script to the genome reference version you desire.
2. This pipeline not only process RNA-seq data from local directory, but also be able to download data from SRA(download .sra file and convert to fastq.gz file) and TCGA(download .bam file and convert to fastq.gz file). Please follow the following instruction to setup the [meta_data/download_list.txt](https://github.com/NYU-BFX/RNA-Seq_Standard/blob/master/meta_data/download_list.txt) file:
   - a. For local stored RNA-seq data in "fastq.gz" format. In the [meta_data/download_list.txt](https://github.com/NYU-BFX/RNA-Seq_Standard/blob/master/meta_data/download_list.txt) file, the first column should be the sample name, the second column shold be the ID, and the third column should be the directory to. the fastq.gz file the files name must follow the following naming convention:
     - ID_L001_R1.fastq.gz (L001 means lane 1, R1 means read 1 for paired-end sequencing)
   - b. For GEO samples from SRA, sample names should be in the first column in [meta_data/download_list.txt](https://github.com/NYU-BFX/RNA-Seq_Standard/blob/master/meta_data/download_list.txt), and the corresponding SRX number should be in the second column and the third column you need to write "SRA".
   - c. For TCGA samples, sample names should be in the first column in [meta_data/download_list.txt](https://github.com/NYU-BFX/RNA-Seq_Standard/blob/master/meta_data/download_list.txt), and the corresponding TCGA UUID of the BAM file should be in the second column and the third column you need to give the path of your TCGA user token file obtained from TCGA for accessing the protected BAM file.
3. In [group information file](https://github.com/NYU-BFX/RNA-Seq_Standard/blob/master/meta_data/group_info.txt) file, you need to categorize your sample into different groups to perform differential expression analysis. You can have multiple [group information file](https://github.com/NYU-BFX/RNA-Seq_Standard/blob/master/meta_data/group_info.txt) with different names. (In this file, the sample name must be identical witht he sample name you use in the first column of the [meta_data/download_list.txt])
4. This pipeline use STAR aligner to align the reads. An transcripts annotation GTF format file ("--sjdbGTFfile" option for STAR aligner in the [params](https://github.com/NYU-BFX/RNA-Seq_Standard/blob/master/params) file) is required for STAR to extract splice junctions and use them to greatly improve the accuracy of the mapping. And this step also counting number of reads per gene for all the genes in the transcripts annotation GTF file using in the alignment step. The downstream RNA-Seq standard report are all based on this transcript annotation GTF.
5. If you have a signature gene list (for example you are doing knockdown of SPOP, then the list should include SPOP and some other related genes) which you would like to check the expression vaule immediately you can define it in the [signature gene list](https://github.com/NYU-BFX/RNA-Seq_Standard/blob/master/meta_data/signature.txt). If you have a list of defined targets (for example some ChIP-Seq experiment define a list of direct targets of your knockdown gene), you can define them in the [target gene list](https://github.com/NYU-BFX/RNA-Seq_Standard/blob/master/meta_data/target.txt). Or you can keep the existing link to the [signature gene list](https://github.com/NYU-BFX/RNA-Seq_Standard/blob/master/meta_data/signature.txt). Please notice the name of the gene in these two file must be consistant with the names in the GTF file you used as "--sjdbGTFfile" option when you are doing STAR alignment.
6. Please change the [params](https://github.com/NYU-BFX/RNA-Seq_Standard/blob/master/params) for any customized parameter settings for all the steps.

**How to run it:**

Once everything has been set up, you can run the pipeline as following:

1. Submit your jobs for each of your sample using the following command if you are using SGE:
2. qsub -b Y -cwd -pe threaded 1 ./run.bash params meta_data/group_info.txt

  And you can change the detailed job scheduling parameter including number of thread used in [code/job_submitter.bash](https://github.com/NYU-BFX/RNA-Seq_Standard/blob/master/code/job_submitter.bash)

1. If you are using another job scheduling system, you may need to write your own job submitter. You can use [code/job_submitter.bash](https://github.com/NYU-BFX/RNA-Seq_Standard/blob/master/code/job_submitter.bash) as a reference. In the end, you need to submit the following master command using your job submitting system to start the whole pipeline.
2. ./run.bash params meta_data/group_info.txt
3. If you have multiple group_info.txt file, you need to run the command for all your group_info.txt files.
4. The summary html report will be generated in [pipeline/summarize](https://github.com/NYU-BFX/RNA-Seq_Standard/blob/master/pipeline/summarize) folder. RNA-seq track files, bam files are in each sample's alignment folder.

**Example of the html report:**

[hESC Report By CellType](http://www.hpc.med.nyu.edu/~gongy05/RNA-Seq_Standard/H1_Cells/By_CellType/By_CellType.html)

[hESC Report By Tissue](http://www.hpc.med.nyu.edu/~gongy05/RNA-Seq_Standard/H1_Cells/By_Tissue/By_Tissue.html)
