# Supplementary material for: lncRNA-screen: an interactive platform for computationally screening long non-coding RNAs in large genomics datasets
Source: BMC Genomics. 2017 Jun 5;18:434. doi: 10.1186/s12864-017-3817-0 (PMC5458484; doi:10.1186/s12864-017-3817-0)
Supplement: Supplementary file 2 — Supplementary material 3. (DOCX 158 kb) [file 12864_2017_3817_MOESM2_ESM.docx]

**SUPPLEMENTARY MATERIAL 3**

**lncRNA-screen**

lncRNA-screen is a comprehensive pipeline for computationally screening putative lncRNA transcripts over large multimodal datasets. It provides a fully automated easy-to-run pipeline which performs data download, RNA-seq alignment, assembly, quality assessment, transcript filtration, novel lncRNA identification, coding potential estimation, expression level quantification, histone mark enrichment profile integration, differential expression analysis, annotation with other type of segmented data (CNVs, SNPs, Hi-C, etc.) and visualization. Importantly, lncRNA-screen generates an interactive report summarizing all interesting lncRNA features including genome browser snapshots and lncRNA-mRNA interactions based on Hi-C data.

**Setting up:**

1. lncRNA-screen has a upstream pipeline which taking care of the standard RNA-Seq analysis. Please follow the instruction from the link below to setup your RNA-Seq analysis pipeline and finish the alignment and assembly step. [RNA-Seq Standard](https://github.com/NYU-BFX/RNA-Seq_Standard). Don't forget to check out the RNA-Seq_Standard HTML report for diagnosis.
   - Attention: lncRNA-screen inherits the "code" and "referenceFiles" directory from RNA-Seq_Standard pipeline. Please make sure you set up RNA-Seq_Standard pipeline folder correctly.
   - If you are using your own RNA-Seq analysis result,
     - You still need to set up "RNA-Seq_Standard" pipeline in order to make "code" and "referenceFiles" accessible for lncRNA-screen.
     - You need to put all the alignment files (one directory per sample) into [inputs/RNA-Seq/pipeline/alignment](https://github.com/NYU-BFX/RNA-Seq_Standard/tree/master/pipeline/alignment) directory. In each sample directory, you need to have a "accepted_hits.bam" file, a "sequencing_info.txt" file (first line contains unstranded/firststrand/secondstrand, second line contains single-end/pair-end) and a ".bw" file which names in sample name.
     - You need to put all the cufflinks generated files (one directory per sample) into [inputs/RNA-Seq/pipeline/cufflinks](https://github.com/NYU-BFX/RNA-Seq_Standard/tree/master/pipeline/cufflinks) directory. In each sample directory, you need to have a "transcripts.gtf" file.
     - You won't be able to get the alignment statistics barplot or featureCounts statistics barplot.
2. Setting up [inputs](https://github.com/NYU-BFX/lncRNA-screen/blob/master/inputs) directory:
   - a. Please replace the [inputs/RNA-Seq](https://github.com/NYU-BFX/lncRNA-screen/blob/master/inputs/RNA-Seq) link with the directory of your RNA-Seq_Standard you set up in step 1.
   - b. Please replace the [inputs/ChIP-Seq](https://github.com/NYU-BFX/lncRNA-screen/blob/master/inputs/ChIP-Seq) link with your ChIP-Seq analysis result. Make sure you have all the peak files in bed format in your [inputs/ChIP-Seq/bed](https://github.com/NYU-BFX/lncRNA-screen/blob/master/inputs/ChIP-Seq/bed) directory. (You can choose to use our ChIP-Seq analysis pipeline if you want from [this link](https://github.com/NYU-BFX/hic-bench/tree/master/pipelines/chipseq-standard).)
   - c. Please follow the example in [inputs/group_info.txt](https://github.com/NYU-BFX/lncRNA-screen/blob/master/inputs/group_info.txt) file to set up your sample sheet. Please make sure you match your sample name with the sample name you used in RNA-Seq_Standard pipeline (Sample name should match the directory name in the RNA-Seq/pipeline/alignment/ folder and RNA-Seq/pipeline/cufflinks).
   - d. Please change the parameters as your desire in [inputs/params.bash](https://github.com/NYU-BFX/lncRNA-screen/blob/master/inputs/params.bash) file.
   - e. [inputs/custom-bashrc](https://github.com/NYU-BFX/RNA-Seq_Standard/blob/master/code/custom-bashrc) file set up the path to all necessary dependencies. If you are not using "Software Environment Management", you need to set up your path to r/3.3.0, python/2.7 and samtools/0.1.19.
   - f. [inputs/job_submitter.bash](https://github.com/NYU-BFX/RNA-Seq_Standard/blob/master/code/job_submitter.bash) is a job submitting tool for SGE system. If you are using another job scheduler, please change it as your desire. But pleas make sure this script take the first parameter as number of thread, and the remaining parameters as the exact command you want to excute at real time in command line.
3. Setting up [referenceFiles](https://github.com/NYU-BFX/lncRNA-screen/blob/master/referenceFiles) directory:
   - a. By default, lncRNA-screen supports hg19 reference genome and use Gencode.v19 as reference annotation. Please run the following command to download and build the reference folder.
   - code/build_referenceFiles.bash
   - b. If you are working on a different reference version or species, please check if you have replaced all the files in this directory as your own desire. Also please make sure you use the same reference version or species in [inputs/RNA-Seq/referenceFiles](https://github.com/NYU-BFX/lncRNA-screen/blob/master/inputs/RNA-Seq/referenceFiles)
   - c. Please don't forget to change your [inputs/params.bash](https://github.com/NYU-BFX/lncRNA-screen/blob/master/inputs/params.bash) file if you use a different reference version or species and finished setting up the [referenceFiles](https://github.com/NYU-BFX/lncRNA-screen/blob/master/referenceFiles) directory.

**How to run it:**

Once everything has been set up, you can run the pipeline in two stages:

1. To finish the analysis, please submit your jobs using the following command if you are using SGE:
2. qsub -b Y -cwd -pe threaded 1 ./run.bash inputs/params.bash inputs/group_info.txt
   - If you are using another job scheduler, you can submit the follwoing command using your own job scheduler:
3. ./run.bash inputs/params.bash inputs/group_info.txt
4. To generate lncRNA snapshots, please run the following command if you are using SGE.
5. cd pipeline/snapshot
6. qsub -b Y -cwd -pe threaded 1 code/batch_lncRNA_snapshot.bash 100 ../coding_potential/lncRNA.bed
   - The "100" is the number of jobs you want to run simultaneously.
   - If you are using another job scheduler, please go to [pipeline/snapshot](https://github.com/NYU-BFX/lncRNA-screen/blob/master/pipeline/snapshot) directory and submit the follwoing command using your own job scheduler:
7. code/batch_lncRNA_snapshot.bash 100 ../coding_potential/lncRNA.bed

**Result:**

The summary html report will be generated in [pipeline/report](https://github.com/NYU-BFX/lncRNA-screen/blob/master/pipeline/report) folder.

Example of html report:

[Roadmap Epigenomics lncRNA Report](http://www.hpc.med.nyu.edu/~gongy05/lncRNA-screen/RoadmapEpigenomics/lncRNA_report.html)

[hESC lncRNA Report](http://www.hpc.med.nyu.edu/~gongy05/lncRNA-screen/H1_Cells/lncRNA_report.html)
