# Supplementary material for: lncRNA-screen: an interactive platform for computationally screening long non-coding RNAs in large genomics datasets
Source: BMC Genomics. 2017 Jun 5;18:434. doi: 10.1186/s12864-017-3817-0 (PMC5458484; doi:10.1186/s12864-017-3817-0)
Supplement: Supplementary file 3 — Supplementary material 4. (DOCX 100 kb) [file 12864_2017_3817_MOESM3_ESM.docx]

**SUPPLEMENTARY MATERIAL 4**

**System Requirement**

|  | Software \| Version \| Included \| Other Setting up work  \|-------------\|---------\|-----------\|--------------------- |
| --- | --- |
|  | samtools \| 1.3 \| No \| Install and set the path |
|  | R \| 3.3.0 \| No \| Install and set the path |
|  | java \| 1.8 \| No \| Install and set the path |
|  | python \| 2.7.3 \| No \| Install and set the path |
|  | perl \| v5.10.1 \| No \| Install and set the path |
|  | STAR \| 2.4.2a \| YES \| None |
|  | Cufflinks \| 2.2.1 \| YES \| None |
|  | Fastqc \| v0.11.3 \| YES \| None |
|  | Picard-Tools \| 2.9.0 \| YES \| None |
|  | featureCoutns \| 1.4.6 \| YES \| None |
|  | sratoolkit \| 2.4.2 \| YES \| None |
|  | gdc-client \| v1.2.1 \| YES \| You may need to recompile the source code(from their github) if running into GLIBC incompatibility issue for CentOS 6.8 or lower version. |
|  | CPAT \| 1.2.2 \| YES \| You may need to recompile from source code following the instruction: http://rna-cpat.sourceforge.net/#installation |

---------------------------------------------------------------------------

---------------------------------------------------------------------------

R session info:

R version 3.3.0 (2016-05-03)

Platform: x86_64-pc-linux-gnu (64-bit)

Running under: CentOS release 6.8 (Final)

locale:

[1] LC_CTYPE=en_US.UTF-8 LC_NUMERIC=C

[3] LC_TIME=en_US.UTF-8 LC_COLLATE=en_US.UTF-8

[5] LC_MONETARY=en_US.UTF-8 LC_MESSAGES=en_US.UTF-8

[7] LC_PAPER=en_US.UTF-8 LC_NAME=C

[9] LC_ADDRESS=C LC_TELEPHONE=C

[11] LC_MEASUREMENT=en_US.UTF-8 LC_IDENTIFICATION=C

attached base packages:

[1] grid tools stats4 parallel stats graphics grDevices

[8] utils datasets methods base

other attached packages:

[1] Gviz_1.16.5

[2] animation_2.5

[3] tidyr_0.6.1

[4] GGally_1.3.0

[5] diagram_1.6.3

[6] shape_1.4.2

[7] preprocessCore_1.34.0

[8] reshape2_1.4.2

[9] hwriter_1.3.2

[10] ReportingTools_2.12.2

[11] knitr_1.15.1

[12] RColorBrewer_1.1-2

[13] pheatmap_1.0.8

[14] DESeq2_1.12.4

[15] TCGAbiolinks_2.0.13

[16] TxDb.Hsapiens.UCSC.hg19.knownGene_3.2.2

[17] GenomicFeatures_1.24.5

[18] AnnotationDbi_1.34.4

[19] ggthemes_3.4.0

[20] EDASeq_2.6.2

[21] ShortRead_1.30.0

[22] GenomicAlignments_1.8.4

[23] SummarizedExperiment_1.2.3

[24] Rsamtools_1.24.0

[25] GenomicRanges_1.24.3

[26] GenomeInfoDb_1.8.7

[27] Biostrings_2.40.2

[28] XVector_0.12.1

[29] IRanges_2.6.1

[30] S4Vectors_0.10.3

[31] BiocParallel_1.6.6

[32] Biobase_2.32.0

[33] BiocGenerics_0.18.0

[34] cowplot_0.7.0

[35] ggplot2_2.2.1

[36] dplyr_0.5.0

loaded via a namespace (and not attached):

[1] R.utils_2.5.0 RSQLite_1.1-2

[3] htmlwidgets_0.8 trimcluster_0.1-2

[5] DESeq_1.24.0 munsell_0.4.3

[7] codetools_0.2-15 colorspace_1.3-2

[9] BiocInstaller_1.22.3 Category_2.38.0

[11] OrganismDbi_1.14.1 supraHex_1.10.0

[13] robustbase_0.92-7 downloader_0.4

[15] TH.data_1.0-8 biovizBase_1.20.0

[17] diptest_0.75-7 R6_2.2.0

[19] doParallel_1.0.10 locfit_1.5-9.1

[21] flexmix_2.3-13 bitops_1.0-6

[23] reshape_0.8.6 assertthat_0.2.0

[25] scales_0.4.1 multcomp_1.4-6

[27] nnet_7.3-12 gtable_0.2.0

[29] affy_1.50.0 ggbio_1.20.2

[31] sandwich_2.3-4 ensembldb_1.4.7

[33] genefilter_1.54.2 GlobalOptions_0.0.11

[35] splines_3.3.0 rtracklayer_1.32.2

[37] lazyeval_0.2.0 acepack_1.4.1

[39] dichromat_2.0-0 hexbin_1.27.1

[41] checkmate_1.8.2 backports_1.0.5

[43] httpuv_1.3.3 Hmisc_4.0-2

[45] RBGL_1.48.1 affyio_1.42.0

[47] gplots_3.0.1 Rcpp_0.12.10

[49] plyr_1.8.4 base64enc_0.1-3

[51] zlibbioc_1.18.0 RCurl_1.95-4.8

[53] rpart_4.1-10 GetoptLong_0.1.6

[55] viridis_0.3.4 zoo_1.8-0

[57] ggrepel_0.6.5 cluster_2.0.5

[59] magrittr_1.5 data.table_1.10.4

[61] dnet_1.0.10 circlize_0.3.10

[63] mvtnorm_1.0-6 whisker_0.3-2

[65] matrixStats_0.52.2 aroma.light_3.2.0

[67] hms_0.3 mime_0.5

[69] xtable_1.8-2 XML_3.98-1.5

[71] mclust_5.2.3 gridExtra_2.2.1

[73] biomaRt_2.28.0 tibble_1.3.0

[75] KernSmooth_2.23-15 R.oo_1.21.0

[77] htmltools_0.3.5 GOstats_2.38.1

[79] Formula_1.2-1 geneplotter_1.50.0

[81] DBI_0.6 matlab_1.0.2

[83] ComplexHeatmap_1.10.2 MASS_7.3-45

[85] fpc_2.1-10 Matrix_1.2-8

[87] readr_1.1.0 parmigene_1.0.2

[89] R.methodsS3_1.7.1 gdata_2.17.0

[91] igraph_1.0.1 coin_1.1-3

[93] foreign_0.8-67 xml2_1.1.1

[95] foreach_1.4.3 annotate_1.50.1

[97] AnnotationForge_1.14.2 rvest_0.3.2

[99] stringr_1.2.0 VariantAnnotation_1.18.7

[101] digest_0.6.12 ConsensusClusterPlus_1.36.0

[103] graph_1.50.0 htmlTable_1.9

[105] dendextend_1.5.2 edgeR_3.14.0

[107] GSEABase_1.34.1 kernlab_0.9-25

[109] shiny_1.0.1 gtools_3.5.0

[111] modeltools_0.2-21 rjson_0.2.15

[113] nlme_3.1-131 jsonlite_1.4

[115] PFAM.db_3.3.0 limma_3.28.21

[117] BSgenome_1.40.1 lattice_0.20-34

[119] httr_1.2.1 DEoptimR_1.0-8

[121] survival_2.40-1 GO.db_3.3.0

[123] interactiveDisplayBase_1.10.3 prabclus_2.2-6

[125] iterators_1.0.8 Rgraphviz_2.16.0

[127] class_7.3-14 stringi_1.1.5

[129] AnnotationHub_2.4.2 latticeExtra_0.6-28

[131] caTools_1.17.1 memoise_1.0.0

[133] ape_4.1

---------------------------------------------------------------------------

---------------------------------------------------------------------------

You are using pip version 7.1.0, however version 9.0.1 is available.

You should consider upgrading via the 'pip install --upgrade pip' command.

argh==0.26.1

argparse==1.2.1

Babel==0.9.4

backports.ssl-match-hostname==3.4.0.2

bayesian-optimization==0.1.0

biom-format==1.1.2

biopython==1.66

boto==2.45.0

bx-python==0.7.1

cas==0.15

chardet==2.2.1

cogent==1.5.3

configobj==4.6.0

Counter==1.0.0

coverage==4.0a6

CPAC==0.3.0

CPAT==1.2.2

CrossMap==0.2.2

csvkit==0.9.0

cutadapt==1.7.1

Cython==0.22

dbf==0.94.3

docutils==0.6

elementtree===1.2.7-20070827-preview

empy==3.1

eta===0.9.8f

ethtool==0.6

firstboot==1.110

foolscap==0.4.2

fpconst==0.7.3

freeipa==2.0.0a0

h5py==1.3.1

HTSeq===0.5.4p3

iniparse==0.3.1

iotop==0.3.2

ipapython==3.0.0

ipdb==0.7

ipython==0.13.2

iwlib==1.0

jdcal==1.0

Jinja2==2.2.1

kerberos==1.1

libgenders==1.2

lockfile==0.9.1

lxml==2.2.3

M2Crypto==0.20.2

MACS==1.4.3

Magic-file-extensions==0.1

matplotlib==1.3.1

memory-profiler==0.31

mercurial==1.4

mglob==0.4

MySQL-python==1.2.3rc1

nest==1.3.0

netaddr==0.7.5

networkx==1.8.1

nibabel==1.3.0

nipype==0.9.2

nose==1.3.1

Numeric==24.2

numexpr==2.4

numpy==1.10.1

openpyxl==2.1.1

ordereddict==1.2

pandas==0.17.1

paramiko==1.7.5

patsy==0.3.0

pexpect==2.3

PIL==1.1.6

Pmw==1.3.2

protobuf==2.4.1

psutil==2.2.1

psycopg2==2.6

pyasn1==0.0.12a0

pycassa==1.9.0

pycrypto==2.0.1

pycurl==7.19.0

Pygments==1.1.1

pygpgme==0.1

pymc==2.3.4

pymol==1.8.0.0

pymongo==2.6.3

pyOpenSSL==0.13.1

pyparsing==2.0.1

pysam==0.8.2.1

python-dateutil==2.4.2

python-default-encoding==0.1

python-dmidecode==3.10.13

python-ldap==2.3.10

python-nss==0.16.0

pytz==2015.7

PyVCF==0.6.7

PyXML==0.8.4

PyYAML==3.10

pyzmq==14.3.1

reportlab==2.3

requests==2.6.0

riak==2.0.2

riak-pb==1.4.4.0

rsa==3.4.1

s3cmd==1.6.1

scdate==1.9.60

scikit-learn==0.16b1

scipy==0.15.1

simplegeneric==0.8

simplejson==3.6.4

six==1.10.0

snp-pipeline==0.3.4

SOAPpy==0.11.6

Sphinx==0.6.6

SQLAlchemy==0.9.7

SSSDConfig==1.13.3

statsmodels==0.6.1

swalign==0.3.3

tables==3.1.1

Theano==0.6.0

thrift==0.9.1

tornado==3.2

traits==4.4.0

Twisted-Core==8.2.0

Twisted-Web==8.2.0

urlgrabber==3.9.1

urllib3==1.10.2

wxPython==2.8.12.0

wxPython-common==2.8.12.0

xlrd==0.9.3

yum-metadata-parser==1.1.2

zope.interface==3.5.2

ZSI==2.0
