# Supplementary material for: lncRNA-screen: an interactive platform for computationally screening long non-coding RNAs in large genomics datasets
Source: BMC Genomics. 2017 Jun 5;18:434. doi: 10.1186/s12864-017-3817-0 (PMC5458484; doi:10.1186/s12864-017-3817-0)
Supplement: Supplementary file 4 — Supplementary material 1. Table S1. Datasets included in this study. Figure S1. featureCounts assigned reads plot: (A) percentages, (B) counts. Figure S2. (A) Principal Component Analysis of Hi-C contact matrices. (B) Average Hi-C count as a function of distance between interacting loci. Figure S3. (A) Distribution of TAD sizes across all the samples. (B) Number of domains across all samples. (C) Pairwise overlaps of TAD boundaries across samples. (DOCX 1349 kb) [file 12864_2017_3817_MOESM4_ESM.docx]

**SUPPLEMENTARY MATERIAL 1**

**lncRNA-screen: an interactive platform for computationally screening long non-coding RNAs in large genomics datasets**

Yixiao Gong^1,2^, Hsuan-Ting Huang^3^, Yu Liang^4^, Thomas Trimarchi^5^, Iannis Aifantis^1,2*^ and Aristotelis Tsirigos^1,2,6*^

**Supplementary Table 1.**  Datasets included in this study.

| adipose-rep1 | large_intestine_fetal_day85_F-rep1 |
| --- | --- |
| adipose-rep2 | large_intestine_fetal_day91_M-rep1 |
| adipose-rep3 | large_intestine_fetal_day98_F-rep1 |
| adrenal_gland_fetal_day101_M-rep1 | liver-rep1 |
| adrenal_gland_fetal_day108_F-rep1 | lung_left_fetal_day105_F-rep1 |
| adrenal_gland_fetal_day108_M-rep1 | lung_left_fetal_day105_M-rep1 |
| adrenal_gland_fetal_day85_F-rep1 | lung_left_fetal_day108_F-rep1 |
| adrenal_gland_fetal_day96_U-rep1 | lung_left_fetal_day91_M-rep1 |
| adrenal_gland-rep1 | lung_left_fetal_day96_M-rep1 |
| adrenal_gland-rep2 | lung_left_fetal_day98_F-rep1 |
| bladder-rep1 | lung-rep1 |
| CD14_primary_cells-rep1 | lung-rep2 |
| CD19_primary_cells-rep1 | lung_right_fetal_day105_F-rep1 |
| CD34_mobilized_primary_cells-rep1 | lung_right_fetal_day105_M-rep1 |
| CD3_primary_cells-rep1 | lung_right_fetal_day96_M-rep1 |
| CD4_primary_cells-rep1 | lung_right_fetal_day98_F-rep1 |
| CD4_primary_cells-rep2 | muscle_arm_fetal_day101-rep1 |
| CD56_primary_cells-rep1 | muscle_arm_fetal_day105_F-rep1 |
| CD8_primary_cells-rep1 | muscle_arm_fetal_day105_M-rep1 |
| CD8_primary_cells-rep2 | muscle_arm_fetal_day120_F-rep1 |
| esophagus-rep1 | muscle_arm_fetal_day120_M-rep1 |
| esophagus-rep2 | muscle_arm_fetal_day127_M-rep1 |
| fibroblasts_skin_abdomen_fetal_day97_M-rep1 | muscle_arm_fetal_day85_F-rep1 |
| fibroblasts_skin_abdomen_fetal_day97_M-rep2 | muscle_arm_fetal_day91_M-rep1 |
| fibroblasts_skin_back_fetal_day96_M-rep1 | muscle_arm_fetal_day96_M-rep1 |
| fibroblasts_skin_back_fetal_day96_M-rep2 | muscle_arm_fetal_day96_M-rep2 |
| fibroblasts_skin_scalp_fetal_day97_M-rep1 | muscle_arm_fetal_day97_M-rep1 |
| fibroblasts_skin_scalp_fetal_day97_M-rep2 | muscle_arm_fetal_day98_F-rep1 |
| gastric-rep1 | muscle_back_fetal_day101_M-rep1 |
| gastric-rep2 | muscle_back_fetal_day105_F-rep1 |
| gastric-rep3 | muscle_back_fetal_day105_M-rep1 |
| H1_BMP4_derived_mesendoderm_cultured_cells-rep1 | muscle_back_fetal_day115_F-rep1 |
| H1_BMP4_derived_mesendoderm_cultured_cells-rep2 | muscle_back_fetal_day127_M-rep1 |
| H1_BMP4_derived_trophoblast_cultured_cells-rep1 | muscle_back_fetal_day85_F-rep1 |
| H1_BMP4_derived_trophoblast_cultured_cells-rep2 | muscle_back_fetal_day91_M-rep1 |
| H1_cell_line-rep1 | muscle_back_fetal_day96_M-rep1 |
| H1_cell_line-rep2 | muscle_back_fetal_day96_M-rep2 |
| H1_cell_line-rep3 | muscle_back_fetal_day98_F-rep1 |
| H1_derived_mesenchymal_stem_cells-rep1 | muscle_leg_fetal_day101_M-rep1 |
| H1_derived_mesenchymal_stem_cells-rep2 | muscle_leg_fetal_day113_F-rep1 |
| H1_derived_neuronal_progenitor_cultured_cells-rep3 | muscle_leg_fetal_day113_M-rep1 |
| H1_derived_neuronal_progenitor_cultured_cells-rep4 | muscle_leg_fetal_day127_M-rep1 |
| heart_aorta-rep1 | muscle_leg_fetal_day96_M-rep1 |
| heart_aorta-rep2 | muscle_leg_fetal_day96_M-rep2 |
| heart_fetal_day120_M-rep1 | muscle_leg_fetal_day96_M-rep3 |
| heart_fetal_day91_F-rep1 | muscle_leg_fetal_day97_M-rep1 |
| heart_left_ventricle-rep1 | muscle_trunk_fetal_day113_F-rep1 |
| heart_left_ventricle-rep2 | muscle_trunk_fetal_day120_F-rep1 |
| heart_right_atrium-rep1 | ovary_fetal-rep1 |
| heart_right_ventricle-rep1 | ovary-rep1 |
| heart_right_ventricle-rep2 | pancreas-rep1 |
| hESC-derived_CD184_P_endoderm_cultured_cells-rep1 | pancreas-rep2 |
| hESC-derived_CD184_P_endoderm_cultured_cells-rep2 | placenta_day113_F-rep1 |
| hESC-derived_CD56_P_ectoderm_cultured_cells-rep1 | psoas_muscle-rep1 |
| hESC-derived_CD56_P_ectoderm_cultured_cells-rep2 | psoas_muscle-rep2 |
| hESC-derived_CD56_P_ectoderm_cultured_cells-rep3 | psoas_muscle-rep3 |
| hESC-derived_CD56_P_ectoderm_cultured_cells-rep4 | sigmoid_colon-rep1 |
| hESC-derived_CD56_P_ectoderm_cultured_cells-rep5 | sigmoid_colon-rep2 |
| hESC-derived_CD56_P_ectoderm_cultured_cells-rep6 | sigmoid_colon-rep3 |
| hESC-derived_CD56_P_mesoderm_cultured_cells-rep1 | skeletal_muscle_lower_limb_fetal_day120_M-rep1 |
| hESC-derived_CD56_P_mesoderm_cultured_cells-rep2 | skeletal_muscle_upper_limb_fetal_day108_F-rep1 |
| HUES64_cell_line-rep2 | small_intestine_fetal_day108_F-rep1 |
| IMR90_cell_line-rep1 | small_intestine_fetal_day108_M-rep1 |
| kidney_fetal_day105_M-rep1 | small_intestine_fetal_day115_M-rep1 |
| kidney_fetal_day108_F-rep1 | small_intestine_fetal_day120_F-rep1 |
| kidney_fetal_day85_F-rep1 | small_intestine_fetal_day91_M-rep1 |
| kidney_fetal_day87_M-rep1 | small_intestine_fetal_day98_F-rep1 |
| kidney_left_fetal_day96_M-rep1 | small_intestine-rep1 |
| kidney_renal_cortex_fetal_day120_F-rep1 | small_intestine-rep2 |
| kidney_renal_cortex_fetal_day91_M-rep1 | small_intestine-rep3 |
| kidney_renal_cortex_fetal_day97_M-rep1 | spinal_cord_fetal_day105_M-rep1 |
| kidney_renal_cortex_left_fetal_day105_M-rep1 | spinal_cord_fetal_day113_F-rep1 |
| kidney_renal_cortex_left_fetal_day105_M-rep2 | spinal_cord_fetal_day96_M-rep1 |
| kidney_renal_cortex_left_fetal_day105_M-rep3 | spleen_fetal_day112_U-rep1 |
| kidney_renal_cortex_left_fetal_day120_M-rep1 | spleen_fetal_day120_M-rep1 |
| kidney_renal_cortex_right_fetal_day105_M-rep1 | spleen-rep1 |
| kidney_renal_cortex_right_fetal_day105_M-rep2 | spleen-rep2 |
| kidney_renal_cortex_right_fetal_day120_M-rep1 | spleen-rep3 |
| kidney_renal_pelvis_fetal_day105_F-rep1 | stomach_fetal_day101_U-rep1 |
| kidney_renal_pelvis_fetal_day91_M-rep1 | stomach_fetal_day105_F-rep1 |
| kidney_renal_pelvis_left_fetal_day105_M-rep1 | stomach_fetal_day107_F-rep1 |
| kidney_renal_pelvis_left_fetal_day105_M-rep2 | stomach_fetal_day108_F-rep1 |
| kidney_renal_pelvis_left_fetal_day105_M-rep3 | stomach_fetal_day110_F-rep1 |
| kidney_renal_pelvis_left_fetal_day120_M-rep1 | stomach_fetal_day127_M-rep1 |
| kidney_renal_pelvis_left_fetal_day97_M-rep1 | stomach_fetal_day98_F-rep1 |
| kidney_renal_pelvis_right_fetal_day105_M-rep1 | stomach_fetal_day98_F-rep2 |
| kidney_renal_pelvis_right_fetal_day105_M-rep2 | testes_pool_fetal_M-rep1 |
| kidney_renal_pelvis_right_fetal_day105_M-rep3 | thymus_fetal_day108_M-rep1 |
| kidney_renal_pelvis_right_fetal_day120_M-rep1 | thymus_fetal_day113_F-rep1 |
| large_intestine_fetal_day105_F-rep1 | thymus_fetal_day115_M-rep1 |
| large_intestine_fetal_day108_M-rep1 | thymus_fetal_day127_M-rep1 |
| large_intestine_fetal_day115_M-rep1 | thymus_fetal_day97_M-rep1 |
| large_intestine_fetal_day120_F-rep1 | thymus_fetal_day98_F-rep1 |

**Supplementary Figure 1.**  featureCounts assigned reads plot: (A) percentages, (B) counts.

**Supplementary Figure 2.** (A) Principal Component Analysis of Hi-C contact matrices. (B) Average Hi-C count as a function of distance between interacting loci.

**Supplementary Figure 3.** (A) Distribution of TAD sizes across all the samples. (B) Number of domains across all samples. (C) Pairwise overlaps of TAD boundaries across samples.
